# Efficacy and safety of YQFM (YiQiFuMai lyophilized injection) on acute ischemic stroke (FAST): rationale and design for a randomized, double-blind, placebo-controlled trial

**DOI:** 10.1186/s12906-025-05036-0

**Published:** 2025-07-22

**Authors:** Yingzhi Xu, Li Sun, Zhaoyou Meng, Xinxing Lai, Dayong Ma, Yuqiu An, Boxuan Du, Qiaosheng Ren, Ying Gao, Kegang Cao

**Affiliations:** 1 Dongzhimen Hospital, Beijing University of Chinese Medicine, Beijing, China; 2 Institute for Brain Disorders, Beijing University of Chinese Medicine, Beijing, China; 3 Department of Neurology, The First Hospital of Jilin University, Jilin, China; 4 Xinqiao Hospital, Army Medical University, Chongqing, China

**Keywords:** Acute ischemic stroke, YiQiFuMai lyophilized injection, Efficacy, Safety, Randomized controlled trial

## Abstract

**Background:**

Low blood pressure at acute ischemic stroke onset is associated with both short- and long-term adverse outcomes. Studies have shown that YQFM (YiQiFuMai lyophilized injection) can ameliorate neurological deficits in ischemic stroke.However, all of these studies are all small-sample clinical observations lacking rigorous study design for AIS with inappropriate blood pressure.

**Aims:**

To describe the design of the YQFM aimed at reducing the disability rate in AIS patients with inappropriate blood pressure.

**Methods:**

This trial is a prospective, multicenter, randomized, double-blind, placebo-controlled, superiority trial aimed at evaluating the efficacy and safety of YQFM in reducing the disability rate in patients with acute hypoperfusion stroke within 90 days. We will recruit 480 patients with AIS within 48 h of symptom onset from 24 hospitals, who have large atherosclerosis, systolic pressure ≤ 155 mmHg, and an NIHSS score of 4–18. Eligible patients will be randomly assigned to receive either YQFM or 0.9% NaCl injection once daily for 10 days and will be followed up until the 90th day after stroke onset.The primary outcome will be the proportion of patients with mRS ≤ 2 at 90 days after patient recruitment. Secondary outcomes will include the proportion of early neurological deterioration at 7 days, patient self-reported outcomes and the syndrome score at 10 days, MMSE scale and BI scale at 90 days.During the trial, adverse events will be recorded. These data will be analyzed according to the predetermined statistical analysis plan.

**Conclusion:**

This study is the first randomized controlled double-blind trial to evaluate the efficacy and safety of YQFM in patients with AIS. This trial will provide evidence-based data for YQFM application in AIS with inappropriate blood pressure.

**Clinical trial registration:**

ChiCTR2300074125 was registered on 31 July, 2023. For more information, please visit Clinical Trials.gov at https://www.chictr.org.cn/showproj.html?Proj=200686.

**Graphical Abstract:**

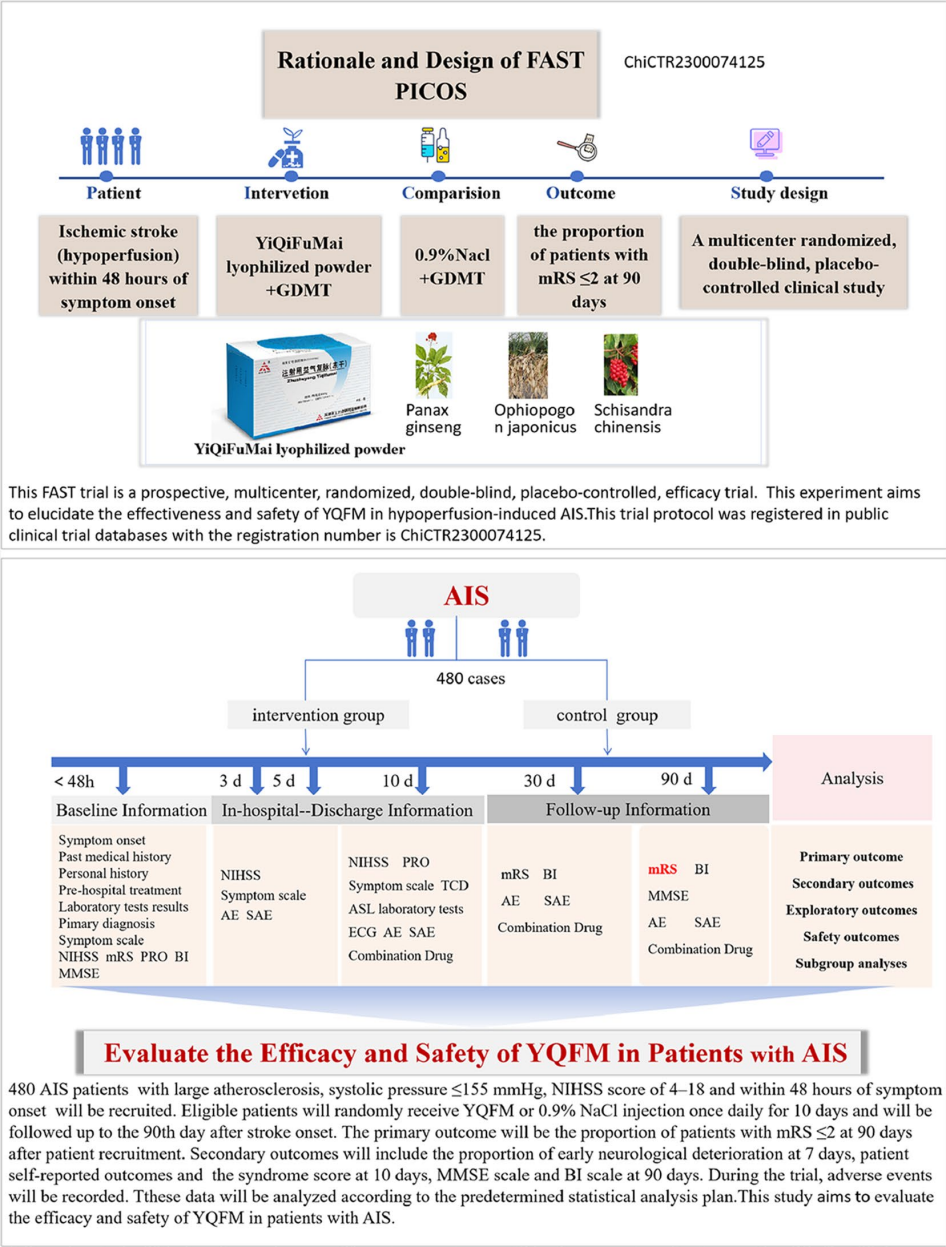

**Supplementary Information:**

The online version contains supplementary material available at 10.1186/s12906-025-05036-0.

## Background

Stroke is a disease characterized by high incidence, high disability and recurrence rates, and frequent complications. Moreover, stroke is the third leading cause of death in the world and the first in China, posing a significant threat to human life [1]. Ischemic encephalopathy affects the quality of life and poses a severe economic burden to patients’ families and society [2–4].

Blood pressure (BP) is often altered in acute stroke [5, 6]. It is well known that extremely high BP is positively associated with adverse clinical outcomes[7, 8]. However, randomized controlled trials on blood pressure lowering have yielded mixed results, hypertensive patients, compared to normotensives, are more likely to have better chances of survival as well as improved cerebral outcomes [9–11]. Previous studies have revealed a “U-shaped” relationship between admission blood pressure and poor outcomes in brain infarction [12–16]. Both low and extremely high blood pressure can lead to poor outcomes [17, 18]. The best prognosis was observed at a systolic blood pressure (SBP) of 160–179 mmHg in the International Stroke Trial (*n* = 17398). In another study, the best prognosis was observed at an SBP of 150–169 mmHg. A SBP between 150 and 200 mmHg represents the values most likely to be associated with surviva [14, 15]. A transition from low to high risk was observed for SBP < 155 mm Hg or for SBP > 220 mm Hg. Patients with SBP < 155 mm Hg were significantly more likely to die within 90 days when compared to those with SBP in the range of 156 to 220 mm Hg [15].

Low SBP in acute ischemic stroke may reduce cerebral perfusion, which may extend the infarction area [19]. Factors like hypotension can cause hemodynamic abnormalities, resulting in insufficient cerebral perfusion pressure, ischemia, and hypoxia of brain tissue. These conditions can lead to neuronal cell death [20–22].

Guidelines from the American Heart Association/American Stroke Association recommend that, for patients with low blood pressure, hypotension and hypovolemia should be corrected to maintain systemic perfusion levels necessary to support organ function. However, no studies have addressed the treatment method of low BP in patients with stroke. Volume expansion therapy has limited effectiveness and increases the burden on the heart, limiting its clinical applications.Clinically, nerve nourishing treatments are often administered, but their effect is not satisfactory [23].

Therefore, it is particularly urgent to explore effective drugs for AIS with inappropriate blood pressure. Alternative or complementary treatment methods have been widely used, and Shengmai preparations have achieved satisfactory therapeutic effects in the treatment of this type of AIS [24, 25]. The use of Shengmai preparations in patients with AIS and low blood pressure has been recommended by the Guideline for the Diagnosis and Treatment of Cerebral Infarction with Integrated Traditional Chinese and Western Medicine (2023).

Currently, the marketed drugs of Shengmai preparations include Shengmai injection and YQFM. YQFM is a lyophilized powder injection, which is convenient for storage and transportation. Therefore, this study selects YQFM as the experimental drug. YQFM [26], a purified extract of Panax ginseng, Ophiopogon japonicus, and Schisandra chinensis, is widely used in the treatment of cardiovascular and cerebrovascular diseases. Research has shown that YQFM can effectively improve the neurological deficit (NFDS) score and daily life index (BI) of patients with acute cerebral infarction [27, 28]. However, these data have been obtained in small-sample clinical observations, and the quality of evidence is relatively low. Therefore, this multicenter, randomized, double-blind, placebo-controlled clinical trial will evaluate the clinical efficacy of YQFM in improving disability in patients with AIS. It aims to obtain high-quality, evidence-based data and improve clinical efficacy in AIS patients with inappropriate blood pressure.

## Methods

### Study design

**Fig. 1:**
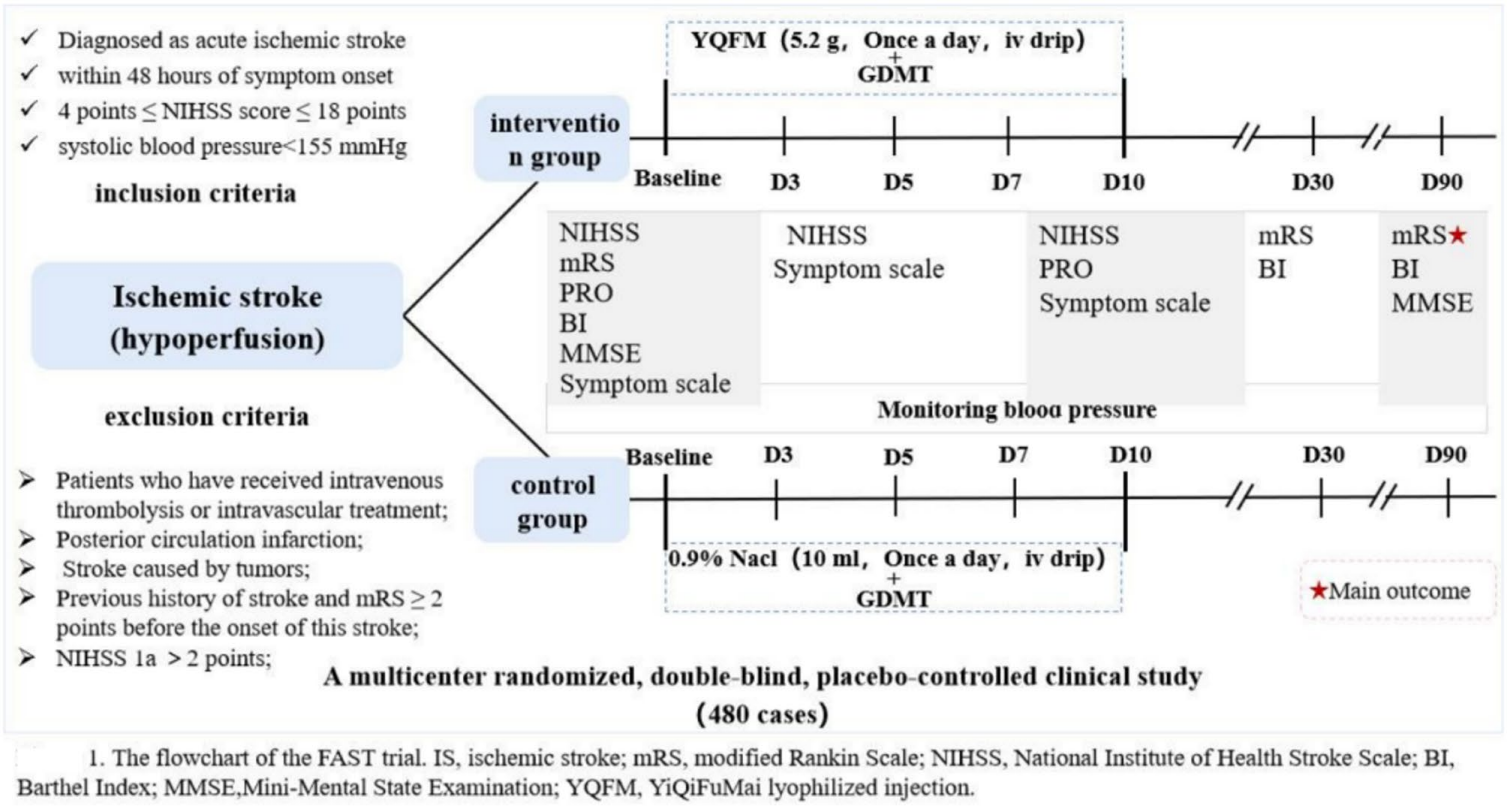
The flowchart of the FAST trial. *Note: Patients’ blood pressure will be measured between 6 and 9 a.m. using the same model of blood pressure monitor. The average of three blood pressure measurements will be recorded

The FAST trial is a prospective, multicenter, randomized, double-blind, placebo-controlled study. This study will enroll 480 subjects. Eligible patients will be assigned to the YQFM group or placebo injection group at a 1:1 ratio and stratified blocked randomization by the study center.The main purpose of the experiment is to test if YQFM is more effective than a placebo in achieving a favorable functional status, defined as an mRS score ≤ 2 at 90 days in patients with AIS. Figure 1 shows the flow diagram of this experiment. The study protocol was described, and a complete checklist is available according to the Standard Protocol Items: Recommendations for Interventional Trials (SPIRIT) guidelines (see Additional File 2 in Supplementary Material) [29].

### Trial population

**Fig. 2:**
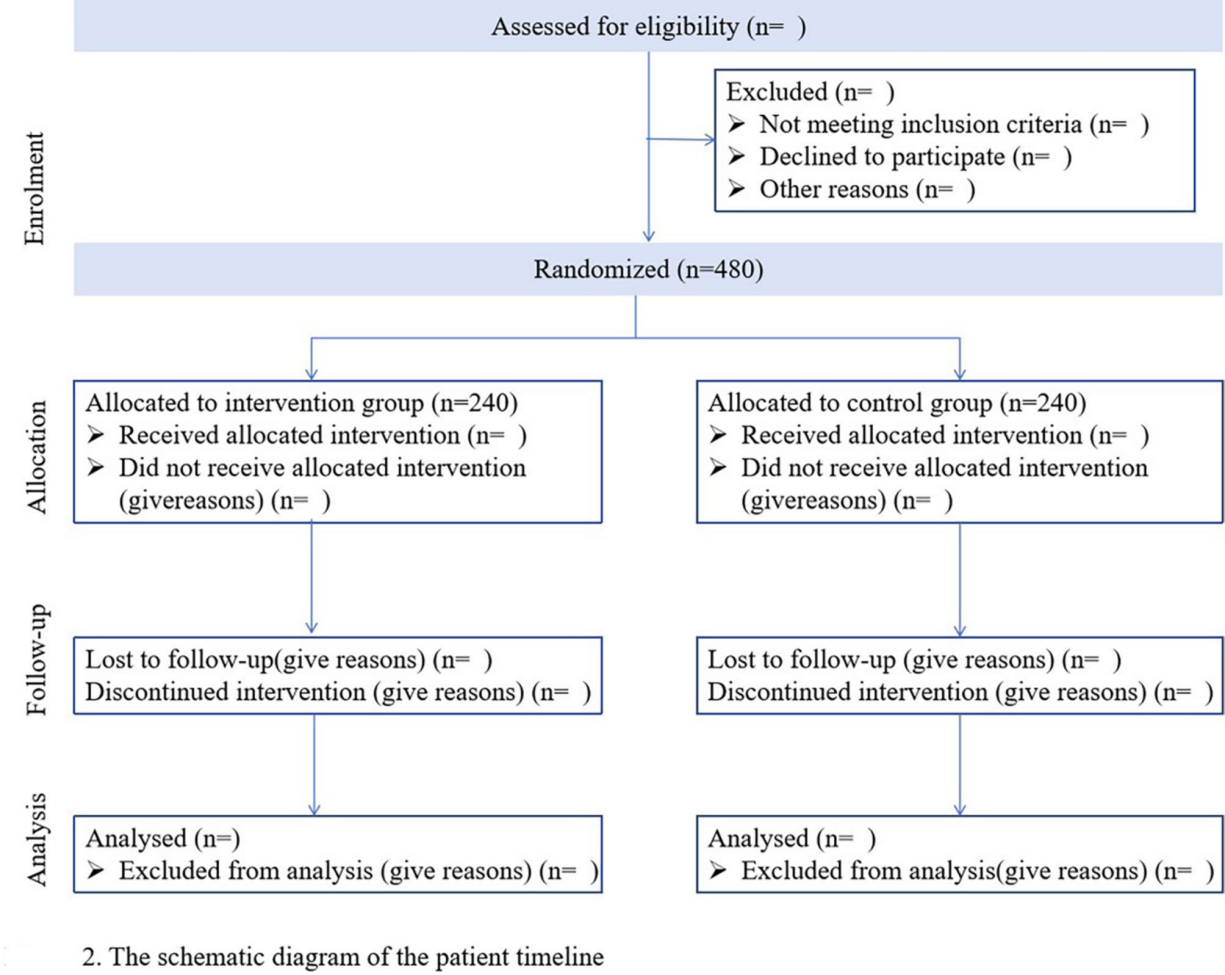
Geographic distribution of participating sites across mainland China

Patients from 24 hospitals in 14 provinces in China were recruited for the study (Fig. 2). The detailed inclusion and exclusion criteria are listed in Table 1.

Recruitment posters will be posted at these hospitals, so that patients can learn about the study and contact researchers. Participants were screened according to screening criteria and informed of the risks and benefits of participating in the study, as well as the collection of blood samples.If patients agree to participate in the study, they will sign an informed consent form. Recruitment began in November 2023, with initial completion expected in December 2025.If there are very serious cases of toxic side effects or insufficient recruitment, the trial will be stopped.

**Table 1:** Inclusion and exclusion criteria

• **Inclusion criteria** • Diagnosed as having acute ischemic stroke; • Large artery atherosclerosis ischemic stroke according to the TOAST classification; • Patients with acute ischemic stroke within 48 h of onset; • Patients aged 18–80 years; • Patients with NIHSS score of 4–18 points; • An average Systolic blood pressure at least 3 times ≤ 155 mmHg before the patient’s enrollment; • Being informed about the study and signing informed consent.	• **Exclusion criteria** • Patients who have received intravenous thrombolysis or intravascular treatment; • Posterior circulation infarction; • Stroke caused by tumors; • Previous history of stroke and mRS ≥ 2 points before the onset of this stroke; • NIHSS 1a > 2 points; • Patients with a yellow and greasy tongue layer; • Patients with other diseases that limit the evaluation of neurological function or affect patient follow-up; • Patients with severe abnormal liver and kidney function with serum ALT, AST, or SCr over two times the upper limit of the reference range; • Have another serious life-threatening illness with life expectancy of less than 3 months; • Pregnant, with recent family planning, or lactating women; • Currently participating in other interventional clinical trials.

### Randomization and blinding method

**Fig. 3:**
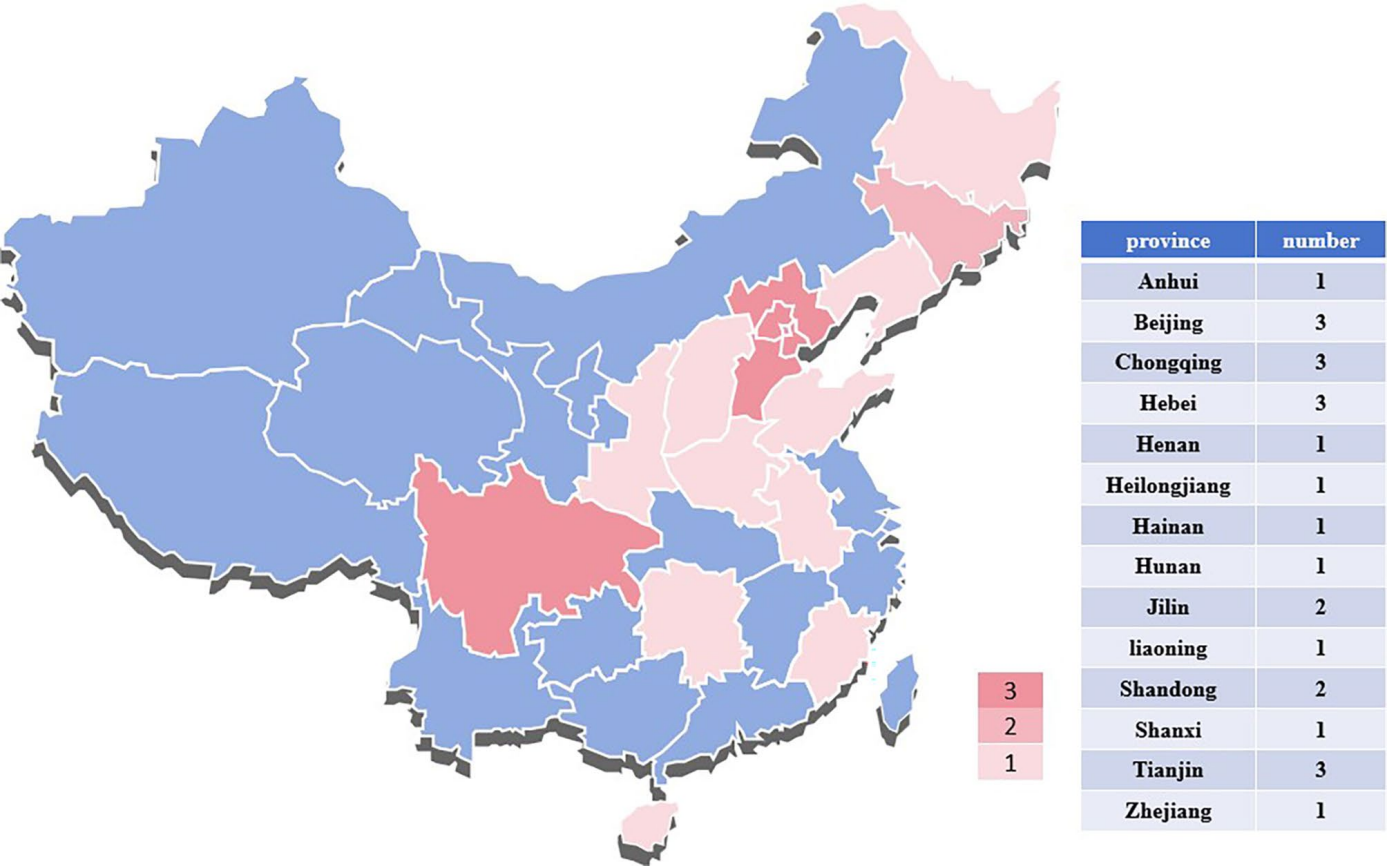
The schematic diagram of the patient timeline

Four hundred and eighty eligible patients with acute ischemic stroke will be randomized to treatment and control groups in a 1:1 ratio using a central randomized approach by investigators within 48 h of symptom onset. Figure 3 shows the flowchart of the included subjects in this study. An independent third-party “Independent Data Committee” will be responsible for the design of randomization schemes and the management of randomization systems.The method of stratified blocked randomization will be utilized to generate a random number grouping table using the professional statistical software PROC PLAN. A random allocation scheme will then be created, with a block size set to 4. It is required that there be 2 treatment groups and 2 control groups in the scheme.

The packaging, weight, and size of the two groups of drugs should be consistent. The allocation sequence and drug coding will be generated by an independent third-party “Independent Data Committee”. The process of drug coding configuration will be recorded in writing and confirmed by the corresponding participants. The researcher of this study will enroll patients strictly based on the order of random allocation table numbers. Researchers will implement randomization by using an online randomization system. They will log into the EDC system to apply for patient randomization codes and drug codes. Afterward, they will submit the requested drug code to the medication administrator.

The researcher and research assistant responsible for enrolling patients, collecting patient clinical information, and assessing outcomes, as well as participants and care providers, are blinded during the research process. Specialized personnel from each unit will be responsible for drug preparation. Dark brown infusion bags and infusion sets will be used for drug infusion to ensure the same appearance of drugs in the two groups.The recovery of relevant infusion supplies and remaining drugs will also be the responsibility of a dedicated person. Throughout the study, the researcher responsible for drug use will record the usage of the investigational drug but will not participate in the collection of other information from the subjects or exchange drug grouping information with others.

### Management and preservation of blind bottoms

The random allocation scheme, selected block length, and random initial seed parameters will be used as blind bases. These blind bases will be configured in the EDC system and stored by the project responsible unit and blinding unit.

### Requirements for unblinding and emergency unblinding

Two levels of unblinding will be employed in this study, with the first level being performed before the statistical analysis and division of the different groups. After the statistical analysis is completed, secondary unblinding will be performed to identify the experimental and control groups. If serious adverse events (SAEs) occur in the clinical trial, and emergency unblinding is required, the main researcher (project leader) will decide whether to proceed with emergency unblinding.

### Subject withdrawal

- Subjects were screened and found to be ineligible based on the enrollment criteria.
- Subjects were not provided experimental medication after enrollment.
- There were no data after randomization.
- During the experiment, the subjects were found to be unfit to continue the experiment. Consequently, the investigator decided to withdraw the cases from the experiment.
- Subjects refused medication and assessment, and as a result, they were lost to follow-up.

### Criteria for discontinuation of tests

- Serious safety issues occurred during the trial, and the investigator believed that the safety of the subjects may be compromised.
- It was observed that the therapeutic effect of the experimental intervention was found to be relatively poor or potentially ineffective, suggesting limited clinical value.
- Major errors were found in the clinical trial protocol, or significant deviations occurred during the trial implementation, making it challenging to assess the trial’s purpose.
- The supervision and administration department requested suspension.

### Interventions

Patients eligible for the study will be assigned to one of the different groups and will receive one of the following two treatments:

- A 5.2 g YQFM injection is diluted into 250 mL of 0.9% sodium chloride injection and administered intravenously once daily for 10 days.
- The placebo injection, which is a 10 mL 0.9% sodium chloride injection, is diluted into 250 mL 0.9% sodium chloride injection and administered through intravenous drip once daily for 10 days.

All enrolled patients will receive guideline-directed medical therapy in accordance with the Chinese guidelines for the diagnosis and treatment of acute ischemic stroke in 2018 [30]. The treatment measures mainly include lipid-lowering, blood sugar-lowering, and antiplatelet aggregation therapies, and it is required that each hospital maintains the same drug varieties when using medications with similar efficacy.On this basis, the test group received YiQiFuMai lyophilized injection and the control group received 0.9% sodium chloride injection for 10 days. The treatment assignment is illustrated in Fig. 1.

Administration of other TCM or Chinese patent medicines, similar in function and indications with the investigational medicine, and of all traditional Chinese medicine injections will be prohibited during the treatment period. Moreover, during the study period, the use of Chinese herbal decoctions (granules) and other traditional Chinese patent medicines for the treatment of acute ischemic stroke will be prohibited.

If an SAE occurs, a participant or their legally authorized representative requests withdrawal from the study, or a participant does not comply with the prescribed intervention, the investigational drug will be discontinued. Any reasons for discontinuing the intervention will be recorded.

### Primary outcome

The main efficacy measure will be the percentage of patients with the ability to engage in “relatively independent” daily living (Modified Rankin Scale ≤ 2, which is defined as relative independence) at D90.

### Secondary outcomes

Secondary outcomes will include the following: the percentage of early neurological deterioration (defined as an increase of 2 or more points in the NIHSS score at D3 compared to baseline), the degree of neurological deficiency (measured by the change in the NIHSS scale between baseline and D10), patient self-reported outcome (measured by the change in the PRO scale between baseline and D10), change in Traditional Chinese medicine syndrome (evaluated using a stroke syndrome elements scale at baseline and D10), degree of disability (evaluated by the distribution of mRS scores at D30 and D90), proportion of patients capable of performing daily living activities (evaluated by BI scale scores at D30 and D90), mental status assessment (evaluated using MMSE scores at D90), recurrence rate of cerebrovascular events and stroke-related mortality within 90 days of onset, and incidence of important vascular events within 90 days.

### Exploratory outcomes

In the intervention and control groups, one-third of the patients from hospitals compliant with brain MRI standards will be observed the change of index of cerebral blood stream and microcirculation under DWI and arterial spin labeling test for brain MRI. Proteomics will be employed to search for differential gene and protein expression.

### Safety outcomes

Safety evaluations include adverse events (AEs), which are any medical events that occur after the subject has received the investigational drug. These events can be manifested as symptoms, signs, diseases, or laboratory abnormalities. It is important to note that AEs may not be causally related to the investigational drug. Additionally, serious adverse events (SAEs) are also evaluated. SAEs include severe outcomes such as death, intracranial hemorrhage, progressive stroke, vascular events.

Laboratory studies (including a complete blood count, routine urine measures, routine stool measures, and measures of liver function, kidney function), vital signs, and an electrocardiogram will be conducted. The causality of adverse events will be determined with reference to the WHO Collaboration Center for International Drug Monitoring, using the criteria of the Uppsala Monitoring Center.

### Monitoring and quality control

Before enrolling the subjects, on-site centralized training, video training, and other forms of training will be conducted for the researchers in each sub-center included in the implementation plan. Each sub-center will issue a researcher folder, which will be saved by a designated person.

A unique login ID will be used by the clinical research coordinator to collect information at each visit point and record data in a timely, accurate, complete, and clear manner in an electronic case report form (eCRF), which will be signed by the researcher. All medical center researchers will make reasonable efforts to follow up patients throughout the entire study period.

A third-party professional monitoring agency will undertake the monitoring tasks of our research group. This agency will regularly monitor all study data and conduct quality control of the study data. Additionally, a clinical research associate will regularly monitor the research data and researcher folder through both online monitoring using an Electronic Data Acquisition System and on-site monitoring.

In this study, inspectors were selected from personnel not directly involved in the experiment. They formulated the inspection process and completed the on-site inspection. At the beginning, middle, and end of the experiment, one third of the research centers were inspected respectively. The inspection focused on the group leader unit, the unit with the fastest and most enrolled subjects, the unit with the first cooperation, and the unit with abnormal data.At the end of the trial, all study centers were inspected at least once.

A third party statistical agency will complete the statistical analysis of the data. Researchers, statistical analysts, monitors, and data managers will design a data verification plan. The data administrator will draft a data audit report and lock the database, which will be carried out jointly by the research team leader and statistical analysts.

### Management of adverse events and adverse reactions

We will record any AEs that occur during the course of the study and will assess whether they are related to the investigational drug, properly handled, and tracked until they are resolved, or until the condition stabilizes. AEs and unexpected events will be reported in a timely manner to the Data Security and Monitoring Board, Ethics Committee, and Chief Investigator.

If AEs occur during the trial, the investigator may take necessary treatment measures according to the condition to ensure the safety of the subjects, record them, and decide whether the subjects’ participation should be terminated in the trial. In case of serious adverse events, the subjects should be withdrawn from the clinical trial, and appropriate measures for the subjects should be taken immediately. The insurance for the enrolled subjects will be covered by this study, ensuring the protection of their rights.

### Sample size calculations

Referring to the distribution of the mRS at the 90 days after stroke onset in the Chinese National Stroke Registry Study, it was found that the proportion of mRS ≤ 2 was 64.6%. In the control group, the percentage of participants with mRS ≤ 2 was 65%, while in the intervention group, the proportion would be 76.5%.The sample size was calculated using PASS 21.0.2.The difference test between the two sample rates was used to calculate the sample size, and a 1:1 design was conducted at the testing level α=0.05 (bilateral) and 1-βß0.8. Considering a 20% dropout rate, each group should include 240 cases, bringing the total number of cases to 480.

$$n = {{2pq{{\left( {z{{\kern 1pt} _\alpha } + {z_\beta }} \right)}^2}} \over {{{\left( {p1 - p2} \right)}^2}}}$$

### Data analysis

The missing data are processed by multiple interpolation. If the primary efficacy index is missing, the previous result is carried forward according to the intentionality analysis.If there is a valid value carried forward after randomization, the last observation is used for carry-over.

The main efficacy measure (the percentage of patients with mRS ≤ 2 ) will be compared and analyzed using the chi-squared test. The statistician will perform covariate adjusted analyses and covariate unadjusted analyses. Subgroup analyses will include sex, age, systolic blood pressure, and the baseline NIHSS score.

For dichotomous outcomes, such as the percentage of BI ≥ 90, distribution of mRS scores, proportion of early neurological deterioration, and recurrence rate of stroke, we will use the chi-squared test or Fisher’s exact test to compare the distribution of patients between the two groups.Logistic regression is utilized to estimate the 95% CI. For continuous variables, including neurological function scores, MMSE scores, PRO scale, cerebral blood flow, such as changes from baseline to treatment endpoints, Student’s t-test or Wilcoxon rank-sum test is employed to analyze the differences between the two groups.Survival data will be estimated using the Kaplan-Meier method, survival curves will be plotted, and efficacy will be evaluated using log-rank tests.

According to the safety set data, statisticians compare and analyze the differences in the incidence of adverse events, major adverse events, and liver and kidney dysfunction, as well as routine blood and urine tests between the two groups. For most safety data, Cox proportional-hazards models are used to estimate the hazard ratios between the two treatment groups.

When 50% of the subjects have completed the study, an interim analysis will be conducted. The continuation or suspension of the study is determined according to the analysis results. If the analysis results indicate that valid results cannot be obtained, the study will be terminated immediately. The study will continue if data analysis suggests that efficacy may be achieved at the end of the study.

## Discussion

Stroke has a significant impact on the physical and mental well-being of patients and is closely linked to high rates of mortality and disability. Additionally, BP tends to rise during the onset of a stroke in order to ensure adequate blood flow to the brain. Previous studies have shown that the optimal range of BP after an ischemic stroke is 155-200mmHg [14], and a BP below this range may lead to more severe brain tissue damage [20, 21]. Improving perfusion is crucial for protecting neurological function and reducing the degree of disability in the treatment of AIS with cerebral tissue hypoperfusion due to inappropriate BP, and there are currently no such therapeutics available.Therefore, it is crucial to discover drugs that can effectively improve perfusion and neurological deficits.

YQFM is manufactured using modern technology. The main ingredients are Panax ginseng, Ophiopogon japonicus, and Schisandra chinensis. This product is widely used in the treatment of acute ischemia cardio-cerebrovascular diseases in China. Studies have reported that YQFM can significantly increase endothelial cell viability and tight junction protein expression, improve blood-brain barrier function [31]. Additionally, YQFM can decrease the mRNA levels of tumor necrosis factor-α, interleukin1β, and interleukin, and can ameliorate the OGD-induced brain microvascular endothelial cell barrier disruption [32]. In addition, YQFM can inhibit excessive autophagy, thereby preventing neuronal damage [33]. YQFM can effectively improve the NFDS score in patients with acute cerebral infarction [27, 28]. However, these data were obtained from a small sample and represent low-quality evidence. At present, YQFM treatment for AIS with inappropriate blood pressure still needs high-quality clinical studies to strengthen the evidence.

Given the current research status, we have designed a large-scale, multicenter, randomized, double-blind, placebo-controlled, parallel-controlled clinical trial to provide higher quality evidence for the safety and efficacy of YQFM in AIS. This study will use the internationally recognized 90-day onset of mRS [34] as the primary outcome measure, with secondary outcome measures including early neurological deterioration, changes in syndrome element scores, daily living activities, MMSE and PRO scale scores. we will evaluate the efficacy and safety of YQFM for the treatment of hypoperfusion-induced cerebral infarction on multiple levels.

Furthermore, this study has two limitations. First, due to funding constraints, we were unable to monitor imaging changes from cranial MRI in each patient. Second, the treatment duration was 10 days, and the follow-up period was 90 days, which is relatively short. These temporal limitations leave the potential impact of YQFM on overall mortality and vascular events uncertain, necessitating further clinical trials with extended follow-up to provide more comprehensive data in this area. In conclusion, the FAST trial aims to deliver high-quality clinical evidence for identifying the target population for YQFM and enhancing clinical efficacy in reducing disability.

## Appendix A. The main ingredients of YQFM

The main ingredients of YQFM were Panax ginseng [Araliaceae; Panax ginseng C.A.Mey.], Ophiopogon japonicus [Asparagaceae; Ophiopogon japonicus (Thunb.)Ker Gawl.], and Schisandra chinensis [Schisandra; chinensis (Turcz.)Baill]. Auxiliary materials were meglumine and mannitol.The specification for each bottle was 0.65 g, which is equivalent to 0.5 g of Panax ginseng, 1.5 g of Ophiopogon japonicus, and 0.75 g of Schisandra chinensis. The bottles were sealed and stored away from direct light. The validity period for the product was 30 months.The drug registration standard number of YQFM is YBZ07062006-2009Z-2015, and the drug approval number, Z20060463, was approved by the State Food and Drug Administration. The batch number of YQFM used in this trial is 20,230,931, which was produced and provided by Tianjin Tasly Pride Pharmaceutical Co., Ltd.YQFM preparations do not contain any restricted drugs or drugs derived from endangered species, and the source of drug materials complies with the code of ethics and national drug administration regulations. More information about YQFM is shown in the Supplementary Material.

## Appendix B. Collection and storage of biological specimens

Biological samples, consisting of one blood specimen and one EDTA-blood specimen, were collected and documented at baseline and 10 days after enrollment. The two blood samples were centrifuged at 3000 rpm at 4 °C for 15 min to separate the plasma and serum, and then stored at -80 °C or in liquid nitrogen. After the study, metabolomics analyses are being performed using the plasma specimens.

## Appendix C. The visit flow chart

**Table:** 

Procedure Visit Window	Screening	Treatment Phase				Follow-up Phase	
		Day 1	Day 3	Day 7 ± 1	Day 10 ± 1	Day 30 ± 3	Day 90 ± 7
History collection		√					
temperature, heart rate and respiratory rate		√	√	√	√		
OCSP	√						
TOAST	√						
Informed consent	√						
BP	BP was measured and recorded daily					√	√
Inclusion/exclusion criteria	√						
Clinical labs (hematology, chemistry, urine)、biological specimens		√			√		
Serum liver and kidney function test	√				√		
Arterial spin labeling(ASL) test for brain MRI		√			√		
Transcranial Doppler(TCD)		√			√		
Cardiac uhrasonography		√					
ECG		√			√		
carotid artery ultrasonography		√					
mRS	√	√				√	√
NIHSS	√		√	√	√		
BI		√				√	√
MMSE		√					√
Ischemic stroke syndrome factor diagnostic scale		√					
Stroke syndrome factor evaluation scale		√	√	√	√		
PRO					√		
medication administration record			√	√	√	√	√
Adverse events			√	√	√	√	√

Note: Information collection of ASL and TCD completed by 8 research centers with ASL check criteria

## Electronic supplementary material

Below is the link to the electronic supplementary material.

Supplementary Material 1

Supplementary Material 2

## Data Availability

No datasets were generated or analysed during the current study.

## Abbreviations

AE: Adverse event
AIS: Acute ischemic stroke
ALT: Alanine aminotransferase
AST: Aspartate aminotransferase
BP: Blood pressure
BI: Barthel index
ECG: Electrocardiogram
eCRF: Electronic case report form
ET: Endothelin
HIF-1α: Hypoxia inducible factor-1α
iNOS: Inducible nitric oxide synthase
MMSE: Mini-mental state examination
mRS: Modified Rankin Scale
NIHSS: National Institute of Health stroke scale
PRO: Patient-reported outcome
ROS: Reactive oxygen species
SAE: SERIOUS adverse event
SCr: Serum creatinine
TCD: Transcranial doppler
YQFM: YiQiFuMai lyophilized injection
