# Supplementary material for: Efficacy and safety of YQFM (YiQiFuMai lyophilized injection) on acute ischemic stroke (FAST): rationale and design for a randomized, double-blind, placebo-controlled trial
Source: BMC Complement Med Ther. 2025 Jul 22;25:284. doi: 10.1186/s12906-025-05036-0 (PMC12285189; doi:10.1186/s12906-025-05036-0)
Supplement: Supplementary file 1 — Supplementary Material 1 [file 12906_2025_5036_MOESM1_ESM.docx]

***Supplementary Files***

**Appendix 1 Standard formulation of Yiqifumai lyophilized powder**

**Appendix 2 Qualitative analysis of chemicals of** **Yiqifumai lyophilized powder**

**Appendix 1 Standard formulation of Yiqifumai lyophilized powder**

| Pinyin name | Complete species name | Authorities/ family name | Scientific name | Proportion(g) |
| --- | --- | --- | --- | --- |
| Hongshen | Panax ginseng | Araliaceae | Panax ginseng C.A.Mey. | 0.50 |
| Maidong | Ophiopogon japonicus | Asparagaceae | Ophiopogon japonicus (Thunb.) Ker Gawl. | 1.50 |
| Wuweizi | Schisandra chinensis | Schisandraceae | Schisandra chinensis (Turcz.) Baill. | 0.75 |

**Appendix 2 Qualitative analysis of chemicals of Yiqifumai lyophilized powder**


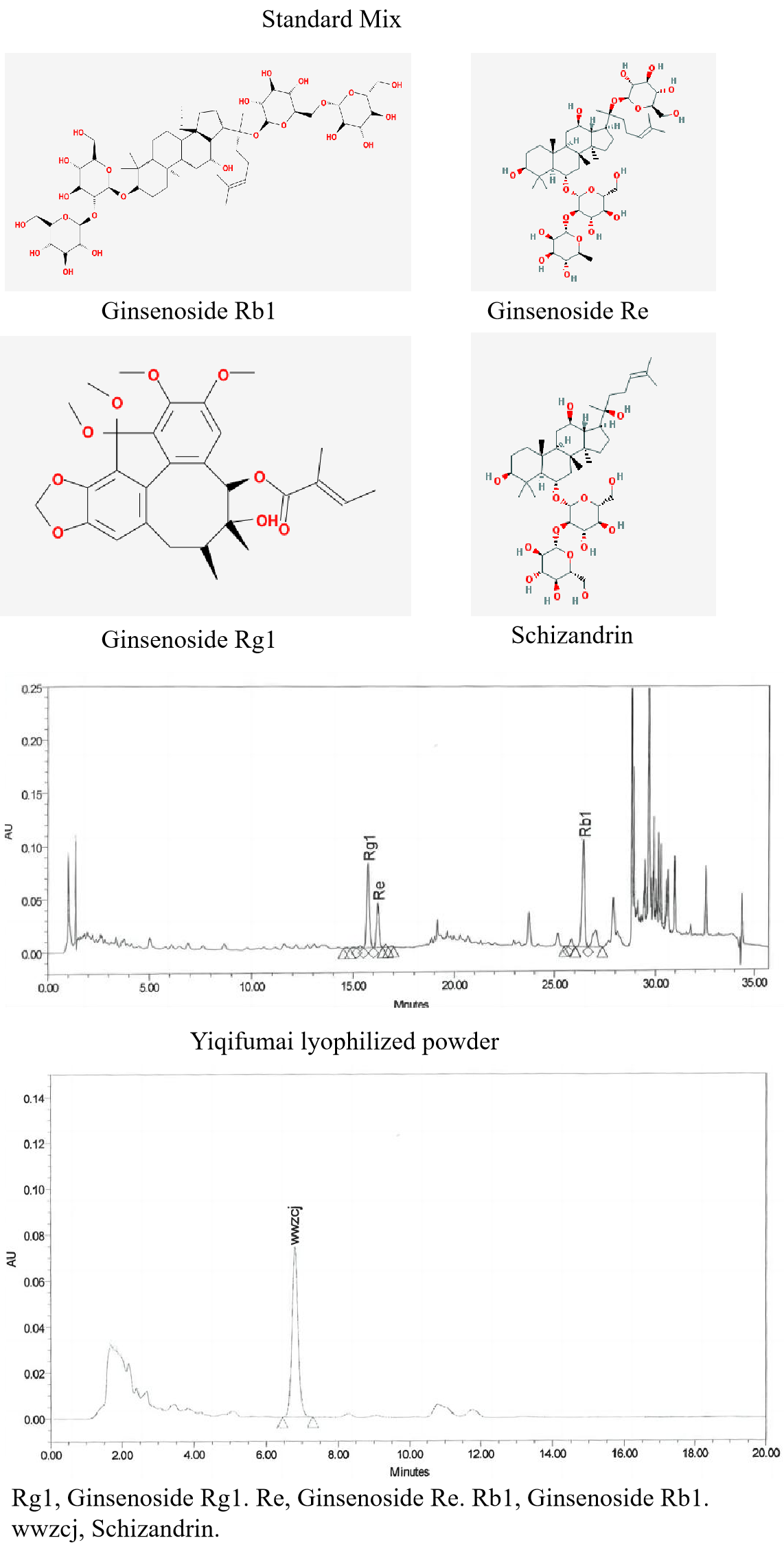


High performance liquid chromatograph (HPLC) of standard mix and Yiqifumai lyophilized powder(YQFM). The HPLC separation was carried out using Ultimate 3000 high performance liquid chromatograph (Thermo Fisher Scientific), equipped with a Inertsil ODS-3 column (4.6mm × 250 mm, 5 µm). The mobile phase A was acetonitrile, and the mobile phase B was 0.05% phosphoric acid solution. The elution gradient was shown as follows:

| Time | The mobile phase A （%） | The mobile phase B（%） |
| --- | --- | --- |
| 0 | 20 | 80 |
| 4.08 | 30 | 70 |
| 6.64 | 35 | 65 |
| 10.21 | 40 | 60 |
| 28.07 | 70 | 30 |
| 30.62 | 95 | 5 |
| 33.18 | 95 | 5 |
| 34.20 | 100 | 0 |
| 36.75 | 100 | 0 |
| 39.30 | 20 | 80 |
| 41.43 | 20 | 80 |

The column temperature was set at 31℃, and the flow rate was set at 0.5 mL / min

Table . Qualitative analysis of chemicals in YQFM

| Chemicals  (PubChem Identifier) | Formula | Retention Time  (min) | Area | Height | EP Plate Court |
| --- | --- | --- | --- | --- | --- |
| Ginsenoside Rb1 | C54H92O23 | 26.44 | 921124 | 100899.50 | 205162.50 |
| Ginsenoside Re | C48H82O18 | 16.22 | 378814 | 42153 | 74165 |
| Ginsenoside Rg1 | C42H72O14 | 15.74 | 738297 | 79833.50 | 66199.50 |
| Schizandrin | C24H32O7 | 6.8 | 895556.34 | 74004 | 7494.50 |
