# Supplementary material for: Efficacy and safety of YQFM (YiQiFuMai lyophilized injection) on acute ischemic stroke (FAST): rationale and design for a randomized, double-blind, placebo-controlled trial
Source: BMC Complement Med Ther. 2025 Jul 22;25:284. doi: 10.1186/s12906-025-05036-0 (PMC12285189; doi:10.1186/s12906-025-05036-0)
Supplement: Supplementary file 2 — Supplementary Material 2 [file 12906_2025_5036_MOESM2_ESM.docx]

***Supplementary Files***

**Reporting checklist for protocol of a clinical trial.**

**Based on the SPIRIT guidelines.**

| **Section/item** | **ltem**  **No.** | **Description** | **Addressed on pagenumber** |
| --- | --- | --- | --- |
| **Administrative information** | | | |
| Title | 1 | Efficacy and Safety of Yiqifumai in the Patients With Acute lschemic Stroke (FAST) Trial: Rationale and design of a prospective, multicenter, randomized, controlled clinical study | Page 1,  line 1 |
| Trial registration | 2a | Clinical trial registration: ChiCTR2300074125 ((July 31, 2023, Clinical Trials.gov: https://www.chictr.org.cn/ showproj.html? Proj =200686). | Page 3,  Line 38 |
|  | 2b | All items from the World Health Organization Trial Registration Data Set | N/A |
| Protocol version | 3 | June 19,2023 | N/A |
| Funding | 4 | This work was supported by the National Key R&D Program of China [grant numbers: 2022YFC3501101. | Page 13 ,  line 370 |
| Roles and responsibilities | 5a | Yingzhi Xu: Conceptualization, Project administration,Writing – original draft and Writing – review & editing. Li Sun: Conceptualization, Investigation and Resources,the acquisition, analysis, and interpretation of data for the work. Zhaoyou Meng: Conceptualization, Investigation and Resources, the acquisition, analysis, and interpretation of data for the work. Xinxing Lai: Conceptualization. Dayong Ma: Conceptualization, Investigation and Resources, the acquisition, analysis, and interpretation of data for the work. Kegang Cao:Conceptualization, Supervision, conception or design of the work, Funding acquisition. Ying Gao:Conceptualization, Supervision, conception or design of the work, Funding acquisition. All authors agree and are responsible for the final manuscript of this paper. | Title Page 13,  Line 373 |
|  | 5b | The sponsor of this clinical trial is Kegang Cao, 86-010-64013209, organization code: 12100000400004449G |  |
|  | 5c | This work was supported by the National Key R&D Program of China [grant numbers: 2022YFC3501101. **Roles and responsibilities**  The sponsor submits study design, collection, management, analysis, and data to funders and is responsible for the interpretation of data writing of the report, publication of the results, writing of the report, and the decision to submit the report for publication.The project management department of the National Key R&D Program of China will organize audit experts to audit the trial every year and submit relevant materials. The process will be independent from investigators and the sponsor. | Title Page 13 ,  line 379 |
|  | 5d | Before enrolling the subjects, on-site centralized training, video training, and other forms of training will be conducted for the researchers in each sub-center included in the implementation plan. Each sub-center will issue a researcher folder, which will be saved by a designated person.  A unique login ID will be used by the clinical research coordinator to collect information at each visit point and record data in a timely, accurate, complete, and clear manner in an electronic case report form (eCRF), which will be signed by the researcher. All medical center researchers will make reasonable efforts to follow up patients throughout the entire study period.  A third-party professional monitoring agency will undertake the monitoring tasks of our research group. This agency will regularly monitor all study data and conduct quality control of the study data. Additionally, a clinical research associate will regularly monitor the research data and researcher folder through both online monitoring using an Electronic Data Acquisition System and on-site monitoring.  In this study, inspectors were selected from personnel not directly involved in the experiment. They formulated the inspection process and completed the on-site inspection. At the beginning, middle, and end of the experiment, one third of the research centers were inspected respectively. The inspection focused on the group leader unit, the unit with the fastest and most enrolled subjects, the unit with the first cooperation, and the unit with abnormal data.At the end of the trial, all study centers were inspected at least once.  A third party statistical agency will complete the statistical analysis of the data.Researchers, statistical analysts, monitors, and data managers will design a data verification plan. The data administrator will draft a data audit report and lock the database, which will be carried out jointly by the research team leader and statistical analysts. | Page 8,  line 219 |
| **Introduction** | | | |
| Background and rationale | 6a | Stroke is a disease characterized by high incidence, high disability and recurrence rates, and frequent complications. Moreover, stroke is the third leading cause of death in the world and the first in China, posing a significant threat to human life.[1] Ischemic encephalopathy affects the quality of life and poses a severe economic burden to patients' families and society.[2­4]  Blood pressure (BP) is often altered in acute stroke.[5­6] It is well known that extremely high BP is positively associated with adverse clinical outcomes.[7­8] However, randomized controlled trials on blood pressure lowering have yielded mixed results, hypertensive patients, compared to normotensives, are more likely to have better chances of survival as well as improved cerebral outcomes.[9­11] Previous studies have revealed a "U-shaped" relationship between admission blood pressure and poor outcomes in brain infarction.[12­16] Both low and extremely high blood pressure can lead to poor outcomes.[17­18] The best prognosis was observed at a systolic blood pressure (SBP) of 160–179 mmHg in the International Stroke Trial (n = 17398). In another study, the best prognosis was observed at an SBP of 150–169 mmHg. A SBP between 150-200 mmHg represents the values most likely to be associated with surviva.[14­15] A transition from low to high risk was observed for SBP <155 mm Hg or for SBP >220 mm Hg. Patients with SBP <155 mm Hg were significantly more likely to die within 90 days when compared to those with SBP in the range of 156 to 220 mm Hg.[15]  Low SBP in acute ischemic stroke may reduce cerebral perfusion, which may extend the infarction area.[19] Factors like hypotension can cause hemodynamic abnormalities, resulting in insufficient cerebral perfusion pressure, ischemia, and hypoxia of brain tissue. These conditions can lead to neuronal cell death.[20­22]  Guidelines from the American Heart Association/American Stroke Association recommend that, for patients with low blood pressure, hypotension and hypovolemia should be corrected to maintain systemic perfusion levels necessary to support organ function. However, no studies have addressed the treatment method of low BP in patients with stroke. Volume expansion therapy has limited effectiveness and increases the burden on the heart, limiting its clinical applications.Clinically, nerve nourishing treatments are often administered, but their effect is not satisfactory.[23]  Therefore, it is particularly urgent to explore effective drugs for AIS with inappropriate blood pressure. Alternative or complementary treatment methods have been widely used, and Shengmai preparations have achieved satisfactory therapeutic effects in the treatment of this type of AIS.[24­25] The use of Shengmai preparations in patients with AIS and low blood pressure has been recommended by the Guideline for the Diagnosis and Treatment of Cerebral Infarction with Integrated Traditional Chinese and Western Medicine (2023).  Currently, the marketed drugs of Shengmai preparations include Shengmai injection and YQFM. YQFM is a lyophilized powder injection, which is convenient for storage and transportation. Therefore, this study selects YQFM as the experimental drug. YQFM,[26] a purified extract of Panax ginseng, Ophiopogon japonicus, and Schisandra chinensis, is widely used in the treatment of cardiovascular and cerebrovascular diseases. Research has shown that YQFM can effectively improve the neurological deficit (NFDS) score and daily life index (BI) of patients with acute cerebral infarction.[27­28] However, these data have been obtained in small-sample clinical observations, and the quality of evidence is relatively low. Therefore, this multicenter, randomized, double-blind, placebo-controlled clinical trial will evaluate the clinical efficacy of YQFM in improving disability in patients with AIS. It aims to obtain high-quality, evidence-based data and improve clinical efficacy in patients with inappropriate blood pressure. | Page 2,  line 43 |
|  | 6b | The placebo injection, which is a 10 mL 0.9% sodium chloride injection, is diluted into 250 mL 0.9% sodium chloride injection and administered through intravenous drip once daily for 10 days. | Page 7  line 170 |
| Objectives | 7 | Therefore, this multicenter, randomized, double-blind, placebo-controlled clinical trial will evaluate the clinical efficacy of YQFM in improving disability in patients with AIS. It aims to obtain high-quality, evidence-based data and improve clinical efficacy in patients with inappropriate blood pressure. | Page 3,  line 82 |
| Trial design | 8 | The FAST trial is a prospective, multicenter, randomized, double-blind, placebo-controlled study. This study will enroll 480 subjects. Eligible patients will be assigned to the YQFM group or placebo injection group at a 1:1 ratio and stratified blocked randomization by the study center. | Page 3 ,  line 88 |
| **Methods: Participants, interventions, and outcomes** | | | |
| Study setting | 9 | Patients from 24 hospitals in 14 provinces in China have recruited for the study. ( Figure 2) | Page 3,  line 101 |
| Eligibility criteria | 10 | **Inclusion criteria**   - Diagnosed as having acute ischemic stroke; - Large artery atherosclerosis ischemic stroke according to the TOAST classification; - Patients with acute ischemic stroke within 48 hours of onset; - Patients aged 18–80 years; - Patients with an the National Institutes of Health Stroke Scale (NIHSS) score of 4–18 points; - An average blood pressure of (at least 3 times) ≤155 mmHg (systolic blood pressure before the patient's enrollment);   Being informed about the study and signing informed consent.  **Exclusion criteria**   - Patients who have received intravenous thrombolysis or intravascular treatment; - Posterior circulation infarction; - Stroke caused by tumors; - Previous history of stroke and modified Rankin Scale (mRS) ≥2 points before the onset of this stroke; - NIHSS 1a >2 points; - Patients with a yellow and greasy tongue layer; - Patients with other diseases that limit the evaluation of neurological function or affect patient follow-up; - Patients with severe abnormal liver and kidney function with serum alanine aminotransferase (ALT), serum aspartate aminotransferase (AST), or serum creatinine (SCr) over two times the upper limit of the reference range; - Have another serious life-threatening illness with life expectancy of less than 3 months; - Pregnant, with recent family planning, or lactating women; - Currently participating in other interventional clinical trials. | Page 4,  line 109 |
| Interventions | 11a | Patients eligible for the study will be assigned to one of the different groups and will receive one of the following two treatments:  A 5.2 g YQFM injection is diluted into 250 mL of 0.9% sodium chloride injection and administered intravenously once daily for 10 days.  The placebo injection, which is a 10 mL 0.9% sodium chloride injection, is diluted into 250 mL 0.9% sodium chloride injection and administered through intravenous drip once daily for 10 days.  All enrolled patients will receive guideline-directed medical therapy (GDMT) in accordance with the Chinese guidelines for the diagnosis and treatment of acute ischemic stroke in 2018,[30] in order to maintain consistency in medication. The treatment assignment is illustrated in Figure 1. | Page 7,  Line 166 |
|  | 11b | **Subject withdrawal**  Subjects were screened and found to be ineligible based on the enrollment criteria.   Subjects were not provided experimental medication after enrollment.   There were no data after randomization.  During the experiment, the subjects were found to be unfit to continue the experiment. Consequently, the investigator decided to withdraw the cases from the experiment.  Subjects refused medication and assessment, and as a result, they were lost to follow-up. | Page 7,  line 150 |
|  | 11c | The recovery of relevant infusion supplies and remaining drugs will also be the responsibility of a dedicated person. Throughout the study, the researcher responsible for drug use will record the usage of the investigational drug but will not participate in the collection of other information from the subjects or exchange drug grouping information with others.  A unique login ID will be used by the clinical research coordinator to collect information at each visit point and record data in a timely, accurate, complete, and clear manner in an electronic case report form (eCRF), which will be signed by the researcher. All medical center researchers will make reasonable efforts to follow up patients throughout the entire study period.  A third-party professional monitoring agency will undertake the monitoring tasks of our research group. This agency will regularly monitor all study data and conduct quality control of the study data. Additionally, a clinical research associate will regularly monitor the research data and researcher folder through both online monitoring using an Electronic Data Acquisition System and on-site monitoring. | Page 5,  Line 133  Page 8,  Line 222 |
|  | 11d | Administration of other TCM or Chinese patent medicines, similar in function and indications with the investigational medicine, and of all traditional Chinese medicine injections will be prohibited during the treatment period. Moreover, during the study period, the use of Chinese herbal decoctions (granules) and other traditional Chinese patent medicines for the treatment of acute ischemic stroke will be prohibited.  If an SAE occurs, a participant or their legally authorized representative requests withdrawal from the study, or a participant does not comply with the prescribed intervention, the investigational drug will be discontinued. Any reasons for discontinuing the intervention will be recorded. | Page 7,  line 179 |
| Outcomes | 12 | Primary outcome  The main efficacy measure will be the percentage of patients with the ability to engage in "relatively independent" daily living (Modified Rankin Scale ≤ 2, which is defined as relative independence) at D90.  Secondary outcomes  Secondary outcomes will include the following: the percentage of early neurological deterioration (defined as an increase of 2 or more points in the NIHSS score at D3 compared to baseline), the degree of neurological deficiency (measured by the change in the NIHSS scale between baseline and D10), patient self-reported outcome (measured by the change in the PRO scale between baseline and D10), change in Traditional Chinese medicine syndrome (evaluated using a stroke syndrome elements scale at baseline and D10), degree of disability (evaluated by the distribution of mRS scores at D30 and D90), proportion of patients capable of performing daily living activities (evaluated by BI scale scores at D30 and D90), mental status assessment (evaluated using MMSE scores at D90), recurrence rate of cerebrovascular events and stroke-related mortality within 90 days of onset, and incidence of important vascular events within 90 days.  Exploratory outcomes  In the intervention and control groups, one-third of the cases will be randomly selected to observe the change of index of cerebral blood stream and microcirculation under DWI and arterial spin labeling test for brain MRI. Proteomics will be employed to search for differential gene and protein expression.  Safety outcomes  Safety evaluations include adverse events (AEs), which are any medical events that occur after the subject has received the investigational drug. These events can be manifested as symptoms, signs, diseases, or laboratory abnormalities. It is important to note that AEs may not be causally related to the investigational drug. Additionally, serious adverse events (SAEs) are also evaluated. SAEs include severe outcomes such as death, intracranial hemorrhage, progressive stroke, vascular events.  Laboratory studies (including a complete blood count, routine urine measures, routine stool measures, and measures of liver function, kidney function), vital signs, and an electrocardiogram will be conducted. The causality of adverse events will be determined with reference to the WHO Collaboration Center for International Drug Monitoring, using the criteria of the Uppsala Monitoring Center. | Page 7,  line 187 |
| Participant timeline | 13 | Please see Figure1. | Page 3,  line 96 |
| Sample size | 14 | Referring to the distribution of the mRS at the 90 days after stroke onset in the Chinese National Stroke Registry Study, it was found that the proportion of mRS ≤2 was 64.6%. In the control group, the percentage of participants with mRS ≤2 was 65%, while in the intervention group, the proportion would be 76.5%.The difference test between the two sample rates was used to calculate the sample size, and a 1:1 design was conducted at the testing level α＝0.05 (bilateral) and 1-β＝0.8. Considering a 20% dropout rate, each group should include 240 cases, bringing the total number of cases to 480.   | Page 9,  line 253 |
| Recruitment | 15 | Patients from 24 hospitals in 14 provinces in China were recruited for the study (Figure 2). The detailed inclusion and exclusion criteria are listed in Table 1.  Recruitment posters will be posted at these hospitals, so that patients can learn about the study and contact researchers. Participants were screened according to screening criteria and informed of the risks and benefits of participating in the study, as well as the collection of blood samples.If patients agree to participate in the study, they will sign an informed consent form. Recruitment began in November 2023, with initial completion expected in December 2025.If there are very serious cases of toxic side effects or insufficient recruitment, the trial will be stopped. | Page 3,  line 101 |
| **Methods: Assignment of interventions (for controlled trials)** | | | |
| **Allocation:** | | | |
| Sequence generation | 16a | Four hundred and eighty eligible patients with acute ischemic stroke will be randomized to treatment and control groups in a 1:1 ratio using a central randomized approach by investigators within 48 hours of symptom onset.Figure 3 shows the flowchart of the included subjects in this study. An independent third-party "Independent Data Committee" will be responsible for the design of randomization schemes and the management of randomization systems.The method of stratified blocked randomization will be utilized to generate a random number grouping table using the professional statistical software PROC PLAN. A random allocation scheme will then be created, with a block size set to 4. It is required that there be 2 treatment groups and 2 control groups in the scheme. | Page 5,  line 113 |
| Allocation concealment mechanism | 16b | The packaging, weight, and size of the two groups of drugs should be consistent. The allocation sequence and drug coding will be generated by an independent third-party "Independent Data Committee". The process of drug coding configuration will be recorded in writing and confirmed by the corresponding participants. The researcher of this study will enroll patients strictly based on the order of random allocation table numbers. Researchers will implement randomization by using an online randomization system. They will log into the EDC system to apply for patient randomization codes and drug codes. Afterward, they will submit the requested drug code to the medication administrator. | Page 5,  Line 121 |
| Implementation | 16c | An independent third-party "Independent Data Committee" will be responsible for the design of randomization schemes and the management of randomization systems.The method of stratified blocked randomization will be utilized to generate a random number grouping table using the professional statistical software PROC PLAN. A random allocation scheme will then be created, with a block size set to 4. It is required that there be 2 treatment groups and 2 control groups in the scheme.  The packaging, weight, and size of the two groups of drugs should be consistent. The allocation sequence and drug coding will be generated by an independent third-party "Independent Data Committee". The process of drug coding configuration will be recorded in writing and confirmed by the corresponding participants. The researcher of this study will enroll patients strictly based on the order of random allocation table numbers. Researchers will implement randomization by using an online randomization system. They will log into the EDC system to apply for patient randomization codes and drug codes. Afterward, they will submit the requested drug code to the medication administrator. | Page 5,  line 115 |
| Blinding (masking) | 17a | The researcher and research assistant responsible for enrolling patients, collecting patient clinical information, and assessing outcomes, as well as participants and care providers, are blinded during the research process. Specialized personnel from each unit will be responsible for drug preparation. Dark brown infusion bags and infusion sets will be used for drug infusion to ensure the same appearance of drugs in the two groups.The recovery of relevant infusion supplies and remaining drugs will also be the responsibility of a dedicated person. Throughout the study, the researcher responsible for drug use will record the usage of the investigational drug but will not participate in the collection of other information from the subjects or exchange drug grouping information with others.  Management and preservation of blind bottoms  The random allocation scheme, selected block length, and random initial seed parameters will be used as blind bases. These blind bases will be configured in the EDC system and stored by the project responsible unit and blinding unit. | Page 5  line 129 |
|  | 17b | Two levels of unblinding will be employed in this study, with the first level being performed before the statistical analysis and division of the different groups. After the statistical analysis is completed, secondary unblinding will be performed to identify the experimental and control groups. If serious adverse events (SAEs) occur in the clinical trial, and emergency unblinding is required, the main researcher (project leader) will decide whether to proceed with emergency unblinding. | Page 6,  line 145 |
| **Methods: Data collection, management, and analysis** | | | |
| Data collection methods | 18a | Before enrolling the subjects, on-site centralized training, video training, and other forms of training will be conducted for the researchers in each sub-center included in the implementation plan. Each sub-center will issue a researcher folder, which will be saved by a designated person.  A unique login ID will be used by the clinical research coordinator to collect information at each visit point and record data in a timely, accurate, complete, and clear manner in an electronic case report form (eCRF), which will be signed by the researcher. All medical center researchers will make reasonable efforts to follow up patients throughout the entire study period. | Page 8,  line 219 |
|  | 18b | A third-party professional monitoring agency will undertake the monitoring tasks of our research group. This agency will regularly monitor all study data and conduct quality control of the study data. Additionally, a clinical research associate will regularly monitor the research data and researcher folder through both online monitoring using an Electronic Data Acquisition System and on-site monitoring. | Page 8,  line 226 |
| Data  management | 19 | A third-party professional monitoring agency will undertake the monitoring tasks of our research group. This agency will regularly monitor all study data and conduct quality control of the study data. Additionally, a clinical research associate will regularly monitor the research data and researcher folder through both online monitoring using an Electronic Data Acquisition System and on-site monitoring.  In this study, inspectors were selected from personnel not directly involved in the experiment. They formulated the inspection process and completed the on-site inspection. At the beginning, middle, and end of the experiment, one third of the research centers were inspected respectively. The inspection focused on the group leader unit, the unit with the fastest and most enrolled subjects, the unit with the first cooperation, and the unit with abnormal data.At the end of the trial, all study centers were inspected at least once.  A third party statistical agency will complete the statistical analysis of the data. Researchers, statistical analysts, monitors, and data managers will design a data verification plan. The data administrator will draft a data audit report and lock the database, which will be carried out jointly by the research team leader and statistical analysts. | Page 8  line 226 |
| Statistical methods | 20a | The missing data are processed by multiple interpolation. If the primary efficacy index is missing, the previous result is carried forward according to the intentionality analysis.If there is a valid value carried forward after randomization, the last observation is used for carry-over.  The main efficacy measure (the percentage of patients with mRS≤2 ) will be compared and analyzed using the chi-squared test. The statistician will perform covariate adjusted analyses and covariate unadjusted analyses. Subgroup analyses will include sex, age, systolic blood pressure, and the baseline NIHSS score.  For dichotomous outcomes, such as the percentage of BI ≥90, distribution of mRS scores, proportion of early neurological deterioration, and recurrence rate of stroke, we will use the chi-squared test or Fisher's exact test to compare the distribution of patients between the two groups.Logistic regression is utilized to estimate the 95% CI. For continuous variables, including neurological function scores, MMSE scores, PRO scale, cerebral blood flow, such as changes from baseline to treatment endpoints, Student's t-test or Wilcoxon rank-sum test is employed to analyze the differences between the two groups.Survival data will be estimated using the Kaplan-Meier method, survival curves will be plotted, and efficacy will be evaluated using log-rank tests.  According to the safety set data, statisticians compare and analyze the differences in the incidence of adverse events, major adverse events, and liver and kidney dysfunction, as well as routine blood and urine tests between the two groups. For most safety data, Cox proportional-hazards models are used to estimate the hazard ratios between the two treatment groups.  When 50% of the subjects have completed the study, an interim analysis will be conducted. The continuation or suspension of the study is determined according to the analysis results. If the analysis results indicate that valid results cannot be obtained, the study will be terminated immediately. The study will continue if data analysis suggests that efficacy may be achieved at the end of the study. | Page 9,  line 262 |
|  | 20b | Subgroup analyses will include sex, age, systolic blood pressure, and the baseline NIHSS score. | Page 9,  line 267 |
|  | 20c | The missing data are processed by multiple interpolation. If the primary efficacy index is missing, the previous result is carried forward according to the intentionality analysis.If there is a valid value carried forward after randomization, the last observation is used for carry-over. | Page 9,  line 262 |
| **Methods: Monitoring** | | | |
| Data monitoring | 21a | A third-party professional monitoring agency will undertake the monitoring tasks of our research group. This agency will regularly monitor all study data and conduct quality control of the study data. Additionally, a clinical research associate will regularly monitor the research data and researcher folder through both online monitoring using an Electronic Data Acquisition System and on-site monitoring.  In this study, inspectors were selected from personnel not directly involved in the experiment. They formulated the inspection process and completed the on-site inspection. At the beginning, middle, and end of the experiment, one third of the research centers were inspected respectively. The inspection focused on the group leader unit, the unit with the fastest and most enrolled subjects, the unit with the first cooperation, and the unit with abnormal data.At the end of the trial, all study centers were inspected at least once.  A third party statistical agency will complete the statistical analysis of the data. Researchers, statistical analysts, monitors, and data managers will design a data verification plan. The data administrator will draft a data audit report and lock the database, which will be carried out jointly by the research team leader and statistical analysts. | Page 8,  line 226 |
|  | 21b | When 50% of the subjects have completed the study, an interim analysis will be conducted. The continuation or suspension of the study is determined according to the analysis results. If the analysis results indicate that valid results cannot be obtained, the study will be terminated immediately. The study will continue if data analysis suggests that efficacy may be achieved at the end of the study. | Page 10,  line 281 |
| Harms | 22 | We will record any AEs that occur during the course of the study and will assess whether they are related to the investigational drug, properly handled, and tracked until they are resolved, or until the condition stabilizes. AEs and unexpected events will be reported in a timely manner to the Data Security and Monitoring Board, Ethics Committee, and Chief Investigator.  If AEs occur during the trial, the investigator may take necessary treatment measures according to the condition to ensure the safety of the subjects, record them, and decide whether the subjects' participation should be terminated in the trial. In case of serious adverse events, the subjects should be withdrawn from the clinical trial, and appropriate measures for the subjects should be taken immediately.The insurance for the enrolled subjects will be covered by this study, ensuring the protection of their rights. | Page 9,  line 242 |
| Auditing | 23 | In this study, inspectors were selected from personnel not directly involved in the experiment. They formulated the inspection process and completed the on-site inspection. At the beginning, middle, and end of the experiment, one third of the research centers were inspected respectively. The inspection focused on the group leader unit, the unit with the fastest and most enrolled subjects, the unit with the first cooperation, and the unit with abnormal data.At the end of the trial, all study centers were inspected at least once. | Page 8,  line 231 |
| **Ethics and dissemination** | | | |
| Research ethics approval | 24 | This trial protocol was reviewed and approved by the Ethics Committee of Dongzhimen Hospital, Beijing University of Chinese Medicine (2023DZMEC492). This clinical trial protocol complies with the relevant provisions of the Declaration of Helsinki and Tokyo for the protection of subjects.Subjects participating in this study will provide written informed consent. During the clinical trial, the personal information of the subject cannot appear in any clinical information.The subject name must be replaced by the number. | Page 12,  line 346 |
| Protocol  amendments | 25 | Any modifications to the protocol which may impact on the conduct of the study, patients’potential benefit or safety will require a formal amendment to the protocol. Such amendment will be agreed upon by sponsor and principal investigator, and approved by the Ethics Committee prior to implementation.Version control will use protocol identifiers and dates, as well as a list of amendments, to help track the history of amendments and identify the most recent protocol version. | Page 13,  line 357 |
| Consent or assent | 26a | Recruitment posters will be posted at these hospitals, so that patients can learn about the study and contact researchers. Participants were screened according to screening criteria and informed of the risks and benefits of participating in the study, as well as the collection of blood samples.If patients agree to participate in the study, they will sign an informed consent form. Recruitment began in November 2023, with initial completion expected in December 2025.If there are very serious cases of toxic side effects or insufficient recruitment, the trial will be stopped. | Page 4,  line 103 |
|  | 26b | NA |  |
| Confidentiality | 27 | During the clinical trial, the personal information of the subject cannot appear in any clinical information.The subject name must be replaced by the number. | Page 12,  line 349 |
| Declaration of interests | 28 | To ensure the impartiality and independence of the project, the Principal Investigator signed the statement of conflict of interest. | Page 13,  line 362 |
| Access to data | 29 | A third party statistical agency will complete the statistical analysis of the data. Researchers, statistical analysts, monitors, and data managers will design a data verification plan. The data administrator will draft a data audit report and lock the database, which will be carried out jointly by the research team leader and statistical analysts. | Page 9  Line 237 |
| Ancillary and  post-trial care | 30 | This trial has purchased insurance for each patient, and if a major serious adverse event occurs during the trial, the insurance company will compensate the patient accordingly. | Page 12,  line 353 |
| Dissemination policy | 31a | Data of this trial will be submitted to the institution designated by the Project Management and the results will be published in publication. | Page 12,  line 355 |
|  | 31b | Those who have materially contributed to the design, conduct, interpretation, and reporting of the clinical trial will be the author of the final trial report. |  |
|  | 31c | The public can access the full trial protocol on the clinical trial registration platform and the data on the stroke disease registration platform. | Page 12,  line 354 |
|  | | | |
| Informed consent materials | 32 | The informed documents were drafted and distributed to the researchers at medical centers, and the informed documents were modified to meet the local ethical requirements.The participants provided their written informed consent to participate in this trial. | Page 12,  line 364 |
| Biological  specimens | 33 | Biological samples, consisting of one blood specimen and one EDTA-blood specimen, were collected and documented at baseline and 10 days after enrollment. The two blood samples were centrifuged at 3000 rpm at 4°C for 15 minutes to separate the plasma and serum, and then stored at -80 °C or in liquid nitrogen. After the study, metabolomics analyses are being performed using the plasma specimens. | Page 11,  line 336 |
